# Community-based wheelchair caster failures call for improvements in quality and increased frequency of preventative maintenance

**DOI:** 10.1038/s41393-021-00689-3

**Published:** 2021-08-19

**Authors:** Anand Mhatre, Jon Pearlman, Mark Schmeler, Benjamin Krider, John Fried

**Affiliations:** Department of Rehabilitation Science and Technology, University of Pittsburgh, Pittsburgh, PA USA

**Keywords:** Disability, Risk factors

## Abstract

**Study design:**

Secondary data analysis of wheelchair failures and service repair logs from a network of wheelchair suppliers.

**Objective:**

To determine the frequency of wheelchair caster failures and service repairs across wheelchair manufacturers and models and investigate the relationships between them.

**Setting:**

Wheelchair caster failures and service repairs occurred in the community.

**Methods:**

Reported caster failure types were classified based on the risk they pose for user injuries and wheelchair damage. Caster failures experienced by users of tilt-in-space and ultralightweight manual wheelchair models and Group 2, 3 and 4 power wheelchair models between January 2017 and October 2019 were analyzed using Chi-Square tests for independence. Correlational analysis of failures and service repairs was performed.

**Results:**

A total of 6470 failures and 151 service repairs reported across four manufacturers and five models were analyzed. Failure types were significantly associated with manufacturers and models, respectively. Users of tilt-in-space wheelchairs, who require greater seating support, experienced twice the proportion of high-risk caster failures than the ultralightweight manual wheelchair users. Similarly, Group 3 and 4 power wheelchair users, who have complex rehabilitation needs, experienced 15-36% more high-risk failures than Group 2 users. Service repairs negatively correlated with high-risk manual wheelchair caster failures.

**Conclusions:**

Wheelchair users who have greater seating and complex rehabilitation needs are at a higher risk for sustaining injuries and secondary health complications due to frequent caster failures. The study findings call for significant reforms in product quality and preventative maintenance practices that can reduce wheelchair failures and user consequences.

## Introduction

Wheelchairs are the primary means of mobility and independence for nearly 70% of people with spinal cord injuries in the United States [1, 2]. However, these assistive devices do not yet meet the user needs fully. The growing research evidence on field evaluation of wheelchairs and laboratory-based testing has shown that manual and power wheelchairs suffer frequent failures [3–5]. Nearly 45-63% wheelchairs in use experience one or more failures and/or repairs in a 6-month period and one-third of the failures result in adverse user consequences, including injuries and bruises [4, 6, 7]. Among wheelchair components, front caster failures account for 27% of all failures [8]. These failures can be risky. For example, caster wheel fractures can cause the wheelchair to tip and the user to fall out of the wheelchair and get hospitalized [4, 8, 9]. Evidence suggests that a user has to wait for an extended period of time for a repair. For example, based on a recent report from the Veterans Health Administration, about 40% of wheelchair repairs took more than a month for completion, during which veterans suffered physical and financial hardships [10]. The report cited a case in which a caster repair required 210 days. Without a functional wheelchair and longer repair times, the user loses access to work, school, and the community and may have to stay in bed. Failures are associated with pressure injuries, hospitalization and reduction in self-perceived health [11]. Consequently, it follows that failures negatively impact wheelchair user’s health and can lead to personal and public health burdens.

Community studies have found that the incidence of wheelchair accidents reduces when preventative maintenance-related service repairs or active checkups are conducted [12, 13]. If maintenance is not performed, a wheelchair user is ten times more likely to have had suffered a wheelchair accident in the past three years [14]. The Centers for Medicare and Medicaid Services (CMS) and private healthcare insurance pays for the repair and replacement of the wheelchair device but do not cover service repairs [15]. The CMS considers general maintenance to be the responsibility of the wheelchair user who usually lacks knowledge, training, tools, ability to repair, and access to replacement parts necessary for maintenance [16]. As a result, service repairs essential for preventative maintenance rarely happen, and users are left vulnerable to wheelchair breakdowns, health consequences, and hardships.

Very few research studies have investigated wheelchair caster failures, the risk they present to a wheelchair user, and mitigation of risk by preventative maintenance [8, 13, 17, 18]. More evidence on the type and variation of caster failures across different wheelchair models is needed. Understanding the frequency of failures that are risky for users can inform design, quality testing standards development, part selection, repair, and maintenance strategies, and reduce the incidence of wheelchair failures and health consequences. This study aims to perform secondary data analysis of community caster failures across manufacturers and models of wheelchairs reported in the Wheelchair Repair Registry (WRR) [19] and explore their relationships. The study also evaluates the differences in caster survivability between manufacturers and the effect of service repairs on caster failure among wheelchair models.

## Methods

### Description of the Wheelchair Repair Registry

The WRR is a wheeled mobility device failure and repair registry developed by the Rehabilitation Engineering Research Center at the University of Pittsburgh from wheelchair repair claims. The claims were reported by repair technicians from a network of wheelchair suppliers using Labor-Tracker, a repair data collection software. Currently, the registry has over 60,000 repairs conducted on more than 5000 wheelchair devices from 25 manufacturers. The devices include 60% power wheelchairs, 35% manual wheelchairs and 5% scooters. The development and structure of the WRR and the description of repairs and failures are published elsewhere [19].

### Data selection and cleaning

Wheelchair models in WRR were assigned Healthcare Common Procedure Coding System (HCPCS) codes found on respective wheelchair order forms [20]. The system of coverage codes like HCPCS is notably used in developed countries but omitted in this manuscript. Manual wheelchair models were named based on their feature or functionality. Power wheelchair models were assigned group numbers depending on the wheelchair configuration listed on wheelchair order forms. For each model, the number of casters (left and/or right) and failures were computed. Caster repairs and failures reported for all manual and power wheelchair manufacturers and their models beginning in January 2017 until October 2019 were selected for data analysis. The analyzed caster failure types were classified based on the associated risks of wheelchair user injury and damage to other wheelchair parts [18]. Caster wheel fracture and bent part were designated as high-risk failures while bearing failure and worn-out tire were designated as low-risk failures. Repairs related to adjustment and lubrication of caster parts were categorized as service repairs. These repairs are part of preventative maintenance [21] and were performed while repairing or replacing another part. Duplicate or missing ticket and failure entries were discarded. Models with 100 caster failures or greater in total were selected for analysis.

### Statistical data analysis

Chi-square tests for independence were conducted to evaluate the relationship between failures and wheelchair models and between failures and manufacturers. Kaplan-Meier survival curves were fitted to high-risk failures (found with casters having purchase date information) and log-rank tests were performed to evaluate differences in survival rates across models. Linear regression analysis was conducted to evaluate the association between service repairs for manual wheelchair models and high-risk failures. Significance was set at *p* < 0.05 and statistical analyses were performed manually.

## Results

A total of 6470 caster failures and 151 service repairs associated with 4 manufacturers and 5 wheelchair models were analyzed. Table 1 includes descriptions of manual and power wheelchair models analyzed in this study. Table 2 shows the distribution of wheelchair caster failures. Manufacturer names are anonymized using M#.

**Table 1:** Wheelchair model descriptions.

Wheelchair Model	Description
*Manual wheelchairs*	
Tilt-in-space wheelchair	Wheelchairs typically prescribed for less-active users who need seating and positioning support
Ultralightweight wheelchair	Wheelchairs typically prescribed for users with active lifestyle and outdoor mobility needs
*Power wheelchairs*	
Group 2	Wheelchairs typically having at most one power seat function and used on mildly uneven terrain
Group 3	Wheelchairs typically having multiple power seat functions and can navigate thresholds, curbs, and other obstacles
Group 4	Wheelchairs typically with all power seat functions and added maneuvering capabilities that are not needed for use in the home

**Table 2:** Distribution of caster failures across wheelchair manufacturers and models.

Wheelchair Model	Manufacturer	Type of Failure				Service Repairs	Manufacturer & Failure Type Relationship
		Wheel Fracture^*^	Bent Part^a^	Bearing Failure^b^	Worn-out Tire^b^		
Tilt-in-space							
	M1	46	17	33	29	15	*Χ*^2^(3, *N* = 344) = 13.84, *p* < 0.05
	M2	104	8	65	42	10	
Ultralightweight							
	M2	119	21	253	92	70	*Χ*^2^(3, *N* = 839) = 15.29, *p* < 0.05
	M3	55	30	206	63	56	
Group 2							
	M4	201	13	338	1053	Not reported	NA
Group 3							
	M1	111	4	121	192		*Χ*^2^(9, *N* = 344) = 58.07, *p* < 0.05
	M2	102	20	161	220		
	M3	55	7	70	126		
	M4	577	16	570	1176		
Group 4							
	M4	55	0	23	76		NA

^a^High-risk caster failures.

^b^Low-risk caster failures.

**Fig. 1:**
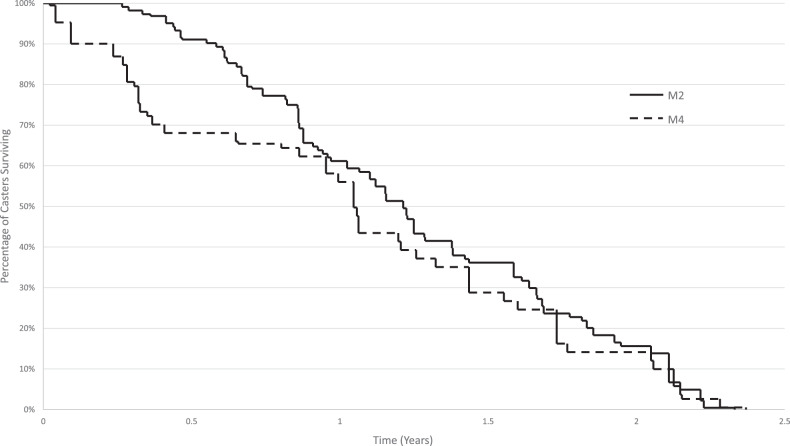
Kaplan-Meier survival curves for high-risk M2 and M4 Group 3 power wheelchair caster failures.

For manufacturer M2, the failures were associated with both the manual wheelchair models, *Χ*^2^(3, *N* = 704) = 42.15, *p* < 0.05. Similarly, for M4, failures were associated with Group 2, 3, and 4 power wheelchairs, *Χ*^2^(6, *N* = 4098) = 207.66, *p* < 0.05. Purchase dates were available for ultralightweight model of M2 and Group 3 models of M2 and M4. Accordingly, survival analyses were carried out with these models. Comparing the proportion of casters surviving at any specific time, significant differences were found between Group 3 wheelchair casters of manufacturers M2 and M4 as shown in Fig. 1, *Χ*^2^(1, *N*_M2 _= 224, *N*_M4 _= 199) = 5.36, *p* < 0.05. Caster service repairs were negatively correlated with high-risk failures of M1, M2, and M3 manual wheelchair casters (*F* (1, 2) = 47.75, *p* < 0.05) with an *R*^2^ of 0.96.

**Fig. 2:**
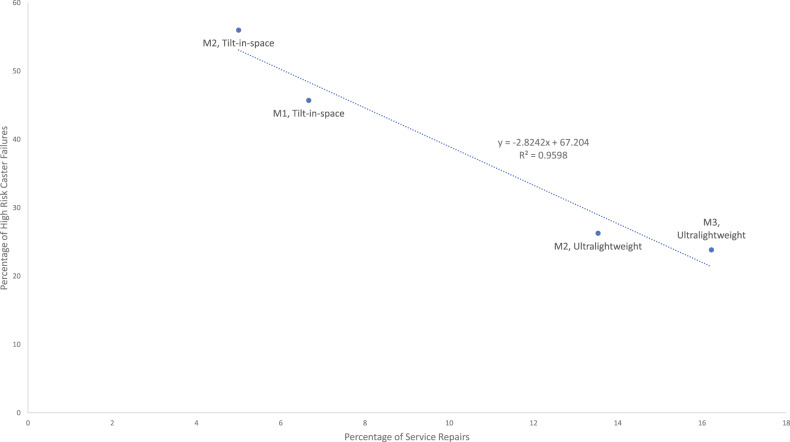
Correlating manual wheelchair caster high-risk failures with service repairs. ^a^Wheelchair manufacturers (M#) and the tilt-in-space and ultralightweight wheelchair models are included in the data callouts.

## Discussion

The proportion of high- and low-risk caster failures are unique to each wheelchair manufacturer and model reported in the WRR. There are two important study findings. First, comparing the proportion of high-risk failures to total failures among manual wheelchairs, the tilt-in-space models encounter nearly twice the high-risk failures than their ultralightweight counterparts. Second, among power wheelchairs, the proportion of high-risk failures to total failures showed a steady rise from 15 to 36% with an increasing group number of the models. These trends suggest that users who require a higher level of seating support and have complex rehabilitation needs are at a greater risk of experiencing caster failures that can cause user injury and other adverse consequences. Users are at additional risk if they use wheelchairs from manufacturers like M4, whose casters break faster than M2 according to the survival curves. Additionally, the high-risk caster failures occur within 1–2 years of wheelchair use, a finding noted in previous community evaluation studies and a common outcome of wheelchair standard testing studies [3, 4, 22]. These study findings call for urgent improvements in caster quality. To raise the quality of casters, the International Society of Wheelchair Professionals (ISWP) and the University of Pittsburgh’s Rehabilitation Engineering Research Center on standards development have developed a caster testing protocol that simulates community failures in the laboratory and screens low-quality casters [9]. This protocol is currently under development for publication as an international wheelchair standard [23]. Based on testing results and iterative evaluations during the design phase, manufacturers can gain feedback to improve caster quality. ISWP has partnered with several national and international manufacturers on caster testing and redesign projects.

Along with high quality, preventative maintenance reduces failures in the community [13, 14], as found in this study. The ultralightweight models were serviced more times than tilt-in-space (see Fig. 2), which could have contributed to a lower high-risk failure count with active users. The regression analysis for manual wheelchairs in Fig. 2 showed that service repairs reduced failure occurrences significantly. This result supports findings from previous wheelchair maintenance studies and highlights the need for preventative maintenance practices and training, especially for the vulnerable users of tilt-in-space wheelchairs [4, 13, 14, 24]. Users should partake in maintenance training programs and educate themselves on using validated maintenance tools [21] to mitigate the risk of breakdowns and consequences. Some service repairs may not be performed owing to the complexity and the lack of tools and capabilities. In such cases, providers shall be incentivized by insurance for carrying out the repairs in collaboration with users and caregivers, perhaps remotely using telehealth approaches.

As per this study, casters incurred rapid fatigue, which made them prone to low- to high-risk failures as early as within a year of use. The rate of fatigue can subside, and early failures can be prohibited if casters do not suffer from road impacts or misuse. Wheelchair skills training [25] enables users, depending on their abilities, to navigate architectural barriers and inaccessible outdoors. For instance, some trained users can wheelie over road bumps and curbs, thus avoiding caster shocks and fatigue. User education on skills and maintenance can prolong the survival of casters and wheelchair parts and prevent failures.

This study demonstrates the significant variability in caster performance across manufacturers and models. This finding should be of particular interest to clinicians, buyers, and insurance—the stakeholders in product selection. It is recommended that stakeholders seek information on standardized testing of products and product performances through reports, publications, and manufacturer specification sheets before selecting a wheelchair and/or wheelchair parts. At the same time, follow-up procedures at regular intervals are essential to understand and possibly document the reliability of prescribed wheelchairs.

One of the highlights of this study is the contrast in survival rates of the same caster models from two manufacturers M2 and M4. Such discrepancies are unwarranted as similar models from different manufacturers are procured at the same price cap, typically in developed countries. This rate increases with increasing group number of power wheelchairs. Group 3 and 4 power wheelchair casters experienced 21% more high-risk failures than Group 2 casters indicating that quality for caster used outdoors is inversely related to product cost. This raises a concern about cost reduction engineering practiced by some manufacturers in the wheelchair industry. Specifically, in the United States, this may result from the policies implemented by the Food and Drug Administration and CMS. Standardized wheelchair testing by independent testing laboratories remains a recommendation and not a requirement for all wheelchairs. Power wheelchair devices are durability tested by manufacturers as per CMS. These practices introduce a risk for bias. Enacting minimum standards as requirements for approval and public disclosure of related materials can ensure that appropriate quality matching the procurement cost is maintained by manufacturers.

This secondary data analysis study uses a larger dataset of wheelchair caster failures reported in the WRR to signify implications for stakeholders involved in wheelchair provision. It is anticipated that stakeholders identify key takeaways on wheelchair quality improvement and preventative maintenance and promote related processes and standards in their practices. On a broader level, regulatory agencies and federal insurance programs can take relevant steps towards informing policy and product procurement based on study outcomes.

### Study limitations

The WRR data lacks data on wheelchair setup, provision, user training, user- or caregiver-led maintenance, user demographic characteristics, technician training, and use conditions that may influence failure type and frequency. Wheelchairs in use that did not encounter caster failures are not included in WRR and were not a part of the data analysis. However, a 45–63% failure rate within 6-months of wheelchair use found in previous studies [4, 6, 7] can be a suitable reference.

### Future work

The future work includes laboratory testing of caster designs reported in the WRR. Comparing the testing results to failure findings reported in this study and communicating design changes to manufacturers is necessary to improve quality and design. Additionally, as the WRR data grows and more failure timepoints and purchase dates become available, it will be possible to reliably compute time to failure for multiple models and wheelchair parts, and inform the frequency of preventative maintenance events.

## Conclusion

Wheelchair caster failures put wheelchair users at risk for multiple consequences, leading to decreased self-perceived health and quality of life. In this study, users who use wheelchair products that provide complex rehabilitation care and greater seating and positioning support were found to experience a greater number of high-risk caster failures. Service repairs are associated with a significant reduction in high-risk caster failures. Improvements in the quality and maintenance of wheelchair products are needed to mitigate the risk of failures and consequences suffered by wheelchair users.

### Data archiving

The data analyzed in this study comes from the WRR hosted at the University of Pittsburgh [19]. The registry may be made available for public access following the completion of the grant.
